# Physical fitness and level of physical activity in adult patients with Marfan syndrome

**DOI:** 10.1186/s13023-025-03869-z

**Published:** 2025-07-07

**Authors:** Madelene Nordeborn, Nanna Brandt, Åsa Cider, Linda Ashman Kröönström

**Affiliations:** 1 Institute of Neuroscience and Physiology, The Sahlgrenska Academy, University of Gothenburg, Gothenburg, Sweden; 2 Occupational and Physical Therapy Department, Sahlgrenska University Hospital/Östra, Gothenburg, Sweden; 3 Adult Congenital Heart Unit, Sahlgrenska University Hospital/Östra, Gothenburg, Sweden

**Keywords:** Marfan syndrome, Adult congenital heart disease, Exercise capacity, Muscle function, Physiotherapy

## Abstract

**Background:**

Marfan syndrome (MFS) is a rare connective tissue disorder adversely affecting several organ systems. This study evaluated the physical fitness and level of physical activity of Swedish adults with MFS with particular reference to age and sex.

**Methods:**

Included were patients with MFS aged ≥ 18 years, registered in the physiotherapy registry at the Adult Congenital Heart Disease unit of Sahlgrenska University Hospital/Östra who had completed at least one physical fitness test.

**Results:**

Of the 1,894 registered patients, 41 met the inclusion criteria, and 17 (42%) were women. Women with MFS had lower exercise capacity (85.8 [32.3] vs. 123.0 [33.4] W, *p* = 0.003) and muscle function (handgrip strength 57.6 [18.8] vs. 117.4 [37.3] lbs, *p* < 0.001; shoulder abduction: 4.4 ± 1.5 vs. 7.4 ± 1.9 kg, *p* < 0.001) than men with MFS. Adults with MFS had significantly lower exercise capacity than reference values (*p* < 0.001). Both men and women with MFS had decreased muscle function (left and right heel lifts: *p* < 0.001 for both; handgrip strength: *p* = 0.021 for women; shoulder abduction: *p* < 0.001 for men; timed-stands test: *p* = 0.007 for women, *p* = 0.009 for men; compared with reference values. Overall, 71% of the patients reached the current physical activity recommendations of 500–1,000 metabolic equivalent of task (MET) minutes per week.

**Conclusions:**

The majority of adults with MFS were sufficiently physically active; however, physical fitness was reduced. Further research is needed to determine whether individualized exercise regimes can improve physical fitness in adults with MFS.

**Supplementary Information:**

The online version contains supplementary material available at 10.1186/s13023-025-03869-z.

## Introduction

Marfan syndrome (MFS) is a rare genetic disorder of the body’s connective tissues [1]. The prevalence of MFS is approximately 0.01–0.02%, which equates to around 1,000–2,000 people living with the disorder in Sweden [2]. The autosomal dominant condition affects several organ systems, including the cardiovascular, respiratory, muscular and skeletal systems, as well as ocular function [1].

MFS is a lifelong and progressive syndrome that affects each individual differently [1]. *FBN1* encodes fibrillin-1, an extracellular matrix protein that is a vital part of the structure of nearly all of the body’s tissues which gives it its elastic properties [3]. A result of reduced connective tissue elasticity is aortic root dilatation with an increased risk of aortic dissection, aneurysm, and rupture [1]. These anomalies are the leading cause of death in adults with MFS; therefore, patients diagnosed with MFS undergo thorough screening of the cardiovascular system [4]. Furthermore, patients with MFS commonly experience both pain and fatigue in their daily lives [5, 6].

One consequence of the effect of MFS on the aortic wall is that patients with MFS have restrictions regarding exercise [7], and many live an inactive life due to fear of exercising [8]. The increased risk of aortic rupture or dissection due to aortic root dilatation has led to recommendations that exercise should be undertaken with caution to avoid mechanical stress on the aortic wall [7]. Physical activity and exercise on a regular basis offer countless benefits to healthy individuals [9] such as increased physical fitness; improved quality of life; and a decreased risk of cardiovascular diseases and other lifestyle related diseases, as well as premature death [9].

To prescribe individualized exercise regimes, the assessment of physical fitness (measured as exercise capacity and muscle function) is essential [10]. Two previous studies have evaluated muscle function in the quadriceps and hamstrings muscles in patients with MFS (thus, in the lower extremity) [11, 12], of which one also assessed maximal exercise capacity on a bicycle [11]. However, research is sparse regarding physical fitness and there is a lack of research regarding tests of muscle function in the upper extremity in patients with MFS. We hypothesized that women would exhibit lower physical fitness compared to men, that increasing age would be associated with reduced physical fitness, and that individuals with MFS would demonstrate lower levels of physical fitness and physical activity relative to healthy reference populations.

In this study, we therefore aimed to evaluate physical fitness and level of physical activity in adults with MFS with particular reference to age and sex, and to compare physical fitness and level of physical activity in adults with MFS to reference values from healthy individuals.

## Methods

### Study design and procedure

This descriptive registry study was conducted at the adult congenital heart disease (ACHD) unit of Sahlgrenska University Hospital/Östra (SU/Ö). Patients with MFS and aortic involvement were assessed by physiotherapists using physical fitness tests and physical activity questionnaires. Patients were informed verbally and in writing about their data being kept in the ACHD registry, and if they accepted, they were asked to provide written informed consent of their approval. Data have been entered into this registry since April 2009 and were retrieved on September 20, 2022, for the purpose of this study.

### Study population

The inclusion criteria were (1) age ≥ 18 years; (2) diagnosis of MFS according to the International Classification of Diseases (ICD) 10 code Q874 (MFS); (3) registration in the physiotherapy registry of the ACHD unit of SU/Ö; and (4) completion of at least one physical fitness test.

**Fig. 1:**
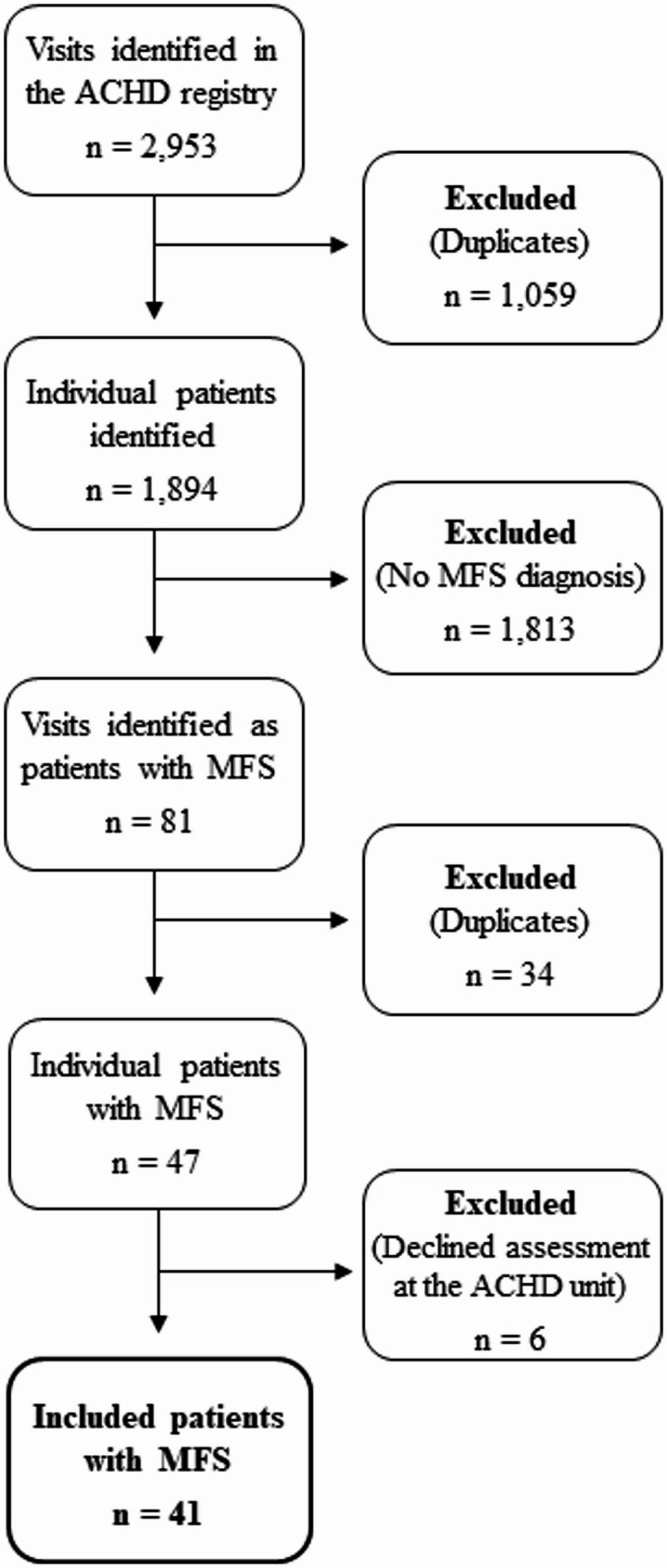
Flow diagram of the patient selection process. ACHD = adult congenital heart disease; MFS = Marfan syndrome; n = number of; Visits = patient visits to the ACHD unit. X-axis shows ratio of patients value and reference value and Y-axis percent of patients included in the study for shoulder abduction and grip strength

A total of 1,894 patients were identified in the ACHD registry. Of these, 47 were identified as patients with MFS, of which 41 met the inclusion criteria and were included in the analysis (Fig. 1). A drop-out analysis comparing the included patients (*n* = 41) with patients who did not undergo physical fitness tests (*n* = 6) showed no significant difference in the sex distribution (male: *n* = 24 [58.5%] vs. *n* = 3 [50%]; *p* = 0.69), New York Heart Association (NYHA) functional class (class I: *n* = 35 [85.4%] and class II: *n* = 6 [14.6%] vs. class I: *n* = 6, [100%]; *p* = 0.32), or age (41.2 [14.5] vs. 34.5 [16.7] years; *p* = 0.21).

### Measurements

The included patients underwent one test to evaluate exercise capacity and five different tests to evaluate isometric (without a noticeable change in muscle length) and isoinertial (refers to the same resistance in the concentric and eccentric phases of muscle contraction) muscle function. Higher values indicate a greater physical capacity, with the exception of the timed-stands test (TST) where the opposite is true. Moreover, they filled out a questionnaire to assess the level of physical activity, as described previously [13].

#### Symptom-limited cycle ergometer test

To evaluate exercise capacity, the patients performed a symptom-limited cycle ergometer test (Monark 828 E, Varberg, Sweden) according to the protocol described by the World Health Organization [14]. This protocol is used in the majority of cardiac rehabilitation centers in Sweden. The test started at a resistance of 25 W, or at 50 W for a few patients, and the resistance was increased by an additional 25 W every 4.5 min. During the test, heart rate was recorded with a heart rate monitor (Polar T31, Bromma, Sweden), and blood pressure was measured manually every 2 min. The patients rated their effort using the Borg Rating of Perceived Exertion (RPE) scale [15]. The test ended when the patient rated their RPE at 15–17 (or in some cases 18). To compare the results of the patients with MFS with the healthy population, Swedish maximal reference values for the cycle ergometer test [16] were used and matched according to the age categories provided in that material.

In the symptom-limited cycle ergometer test, for patients with MFS who did not complete the full 4.5 min (or 5 min) at their final wattage, the values were calculated according to the method of Strandell [17]. Calculations were performed to enable comparisons between the symptom-limited cycle ergometer test and the maximal cycle ergometer test. Specifically, differences in the RPE at which patients ended their tests were corrected, and two equations were applied to obtain a value that was comparable to the maximal value [18].

#### Unilateral isoinertial shoulder flexion

The patients were seated on a stool with their back against the wall and a weight in their dominant hand. Male patients performed the test with a 3-kg weight and female patients with a 2-kg weight. Patients were instructed to repeatedly elevate the testing arm from 0° to 90° flexion in sync with a metronome (20 contractions/min) until failure. The reliability of unilateral isoinertial shoulder flexion to assess muscular endurance in both healthy individuals and patients with chronic heart failure is considered good [19].

#### Unilateral isoinertial heel lift

The heel lift test was performed using a 10° wedge placed in front of a wall. Before starting, adjustments were made to ensure the patients could perform the heel lifts with full range of motion. The patients were required to touch the measuring stick with the top of their head. Patients put one foot on the wedge with the other held up above the floor, and they were allowed to touch the wall for balance purposes. Tests were performed unilaterally, but both legs were tested. Heel lifts were performed in sync with a metronome (one heel lift every other second) until failure, and the maximum number of lifts for each leg were registered. To compare the results of patients with MFS to healthy individuals, reference values for healthy individuals aged 20–80 years old [20] were used. The reliability of unilateral isoinertial heel lift in healthy individuals is considered good [20].

#### Isometric handgrip strength

To evaluate isometric handgrip strength, the hydraulic dynamometer Jamar^®^ (Sammons Preston, Rolyan, Chicago, IL, USA) was used. The patient sat on a chair with the shoulder adducted, elbow at 90°, and forearm fully resting on a surface. Only the patient’s dominant hand was tested. The test was performed three times, and the mean value was calculated. To compare the results of patients with MFS with those of healthy individuals, reference values for healthy individuals were used [21]. When using a standardized protocol for testing handgrip strength and performing the test three times, interrater reliability and test-retest reliability are high [22].

#### Timed-stands test

The TST was used to evaluate functional power in the lower extremities. The test began with the patient sitting on a stool or chair without armrests. The patient’s arms were placed across their chest with both feet touching the ground, either with shoes or barefoot. The patient was instructed to stand up and sit down 10 times at their maximal capacity and speed and the time was measured to the nearest tenth of a second [23, 24]. The results were compared with reference values from healthy individuals aged 20–85 years [23]. The validity and reliability of the TST for assessing lower-extremity function are considered good [24].

#### Unilateral isometric shoulder abduction

The test was performed with the portable isometric dynamometer IsoForce Control^®^ (Medical Device Solutions, Oberburg, Switzerland). The patient was seated on a stool with their back against the wall, legs extended with one leg over the other, and one heel on the floor. Only the patient’s dominant arm was tested. A strap was placed around the wrist, and the arm was elevated to 90° in scaption. On command, the patient was instructed to pull straight upwards to their maximum capacity. The test was performed three times, and the mean value was calculated. The results were compared with reference values from healthy individuals aged 19–57 years [25]. Both intra–rater reliability and inter–rater reliability are considered high when using an isometric dynamometer to assess shoulder strength [26].

#### International physical activity Questionnaire - Short form

The International Physical Activity Questionnaire - Short Form (IPAQ-SF) [27] was used to assess the patients’ level of physical activity during their day-to-day lives. It is a self-reported form consisting of seven items with open-ended questions. The questionnaire was developed based on a target population aged 15–69 years, and the patients answered the questions regarding their activity over the past 7 days. The IPAQ-SF gives an indication of the patient’s level of activity, which is graded as low, moderate, or high depending on the amount of time spent being physically active and the metabolic equivalent of task (MET) min/week. The results were compared with the healthy population using reference values for IPAQ categories [28] and MET min/week [29].

### Statistical methods

The statistical software program Statistical Package for Social Sciences (SPSS^®^) 28.0 (IBM Corp., Armonk, NY, USA) was used for all statistical analyses. The age groups used for the analyses were 18–29, 30–49, and 50–69 years.

The distribution of the variables was evaluated using quantile–quantile plots. Normally distributed continuous data are reported as the mean (standard deviation, SD) and non-normally distributed continuous data are reported as the median [interquartile range, IQR]. If *n* ≤ 3 no IQR is presented. IPAQ data were calculated according to the scoring manual. Normally distributed data were compared between two independent groups, using the Student’s t-test, while non-normally distributed data was compared between two independent groups using the Mann–Whitney U test. For ordinal variables, the chi-square test was used. When comparing more than two independent groups, one-way analysis of variance (ANOVA) was used for normally distributed data, and the Kruskal–Wallis test was used for non-normally distributed data. When comparing the results of patients with MFS with reference values from healthy individuals, the one sample t-test was used for normally distributed data, the one sample Wilcoxon signed rank test was used for non-normally distributed data, and the chi-square test was used for ordinal variables. A significance level of *p* < 0.05 was considered statistically significant for all analyses.

## Results

### Patient selection and demographics

The patients were aged between 18 and 69 years at the time of the assessment, with a mean age of 35 (14.3) years. Of the included patients, 17 (42%) were women (*n* = 17). Overall, 12 patients (29%) had undergone corrective cardiac procedures, some multiple times (Table 1).

**Table 1:** Demographic data of the included patients

	All patients *n* = 41 (100%)	Women *n* = 17 (42%)	Men *n* = 24 (68%)
Age groups			
18–29 years	18 (44)	7 (41)	11 (46)
30–49 years	16 (39)	7 (41)	9 (37)
50–69 years	7 (17)	3 (18)	4 (17)
Age, years, mean (SD)	35 (14.3)	37 (14.8)	34 (14.4)
Height, m, mean (SD)	1.88 (0.1)	1.77 (0.1)	1.95 (0.1)
Weight, kg, mean (SD)	79.1 (16.2)	69.1 (13.9)	86.3 (13.9)
BMI, kg/m^2^, mean (SD)	22.4 (3.6)	22.1 (3.8)	22.6 (3.5)
Secondary cardiac diagnosis			
Left ventricular failure	1 (2.4)	1 (5.9)	0 (0)
Aortic root dilatation, dissected aortic aneurysm, aortic dissection	5 (12.2)	3 (17.6)	2 (8.3)
Thoracic aortic aneurysm, non-dissected	6 (14.6)	0 (0)	6 (25)
Mitral valve insufficiency	3 (7.3)	3 (17.6)	0 (0)
Ascending aorta dilatation	13 (31.7)	6 (35.3)	7 (29.2)
Heart medications	30 (73)	13 (76)	17 (71)
Beta-blockers	22 (54)	11 (65)	11 (64)
ACEi, ARB	12 (40)	5 (38)	7 (41)
Warfarin, Aspirin	9 (30)	3 (23)	6 (35)
Diuretic	1 (2)	0 (0)	1 (4)
Aldosterone	2 (5)	2 (12)	0 (0)
Digitalis	2 (5)	2 (12)	0 (0)
Other	8 (20)	4 (24)	4 (17)
NYHA			
NYHA I	35 (85)	14 (82)	21 (88)
NYHA II	6 (15)	3 (18)	3 (12)
NYHA III/IV	0 (0)	0 (0)	0 (0)
Corrective cardiac procedures*	12 (29.2)	3 (17.6)	9 (37.5)
Sternotomy	11 (26.8)	1 (5.9)	10 (41.7)
Thoracotomy	2 (4.8)	2 (11.8)	1 (4.2)
Catheterization	1 (2.4)	0 (0)	1 (4.2)
Smoking			
Smokers	3 (7)	1 (6)	1 (8)
Ex-smokers	5 (12)	4 (27)	1 (4)
Non-smokers	33 (81)	12 (70)	21 (88)

The data are presented as n (%) unless otherwise stated. BMI = body mass index; ACEi = angiotensin converting enzyme inhibitor; ARB = angiotensin receptor blocker; NYHA = New York Heart Association (functional classification)

*The total number is not equivalent to the sum of procedures because of some patients having undergone multiple procedures

### Physical fitness

Supplementary Fig. 1 depicts participation in tests of physical fitness. When comparing men and women with MFS, there was a significant difference in submaximal exercise capacity (*p* = 0.003) and muscle function (shoulder flexion: *p* = 0.007; right heel lifts: *p* = 0.022; left heel lifts: *p* = 0.015; handgrip strength: *p* < 0.001; shoulder abduction: *p* < 0.001; TST: *p* = 0.010) in favour of men (Table 2). Furthermore, in women with MFS there was a significant difference in shoulder flexion between the age groups (18–29 years: 22 (9.7) kg; 30–49 years: 30 (9.2) kg; 50–69 years: 10 (3.1) kg; *p* = 0.016) (Supplementary Table 1).

**Table 2:** Tests of physical fitness and physical activity in patients with Marfan syndrome by sex

	All patients *n* = 41	Women *n* = 17	Men *n* = 24	*p*
**Cycle test**				0.003^a^
Submaximal, W,	109.2 (37.3)	85.8 (32.3)	123.0 (33.4)	
n (%)	35 (85)	13 (76)	22 (92)	
**Muscle function**				
Shoulder flexion, repetitions	32.1 (18.4)	23.1 (11.0)	38.5 (20.0)	0.007^a^
n (%)	41 (100)	17 (100)	24 (100)	
Heel lift R,	16.6 (8.5)	12.7 (5.5)	19.3 (9.2)	0.022^a^
repetitions n (%)	34 (83)	14 (82)	20 (83)	
Heel lift L, repetitions	18.1 (9.1)	13.6 (9.9)	21.2 (9.8)	0.015^a^
n (%)	34 (83)	14 (82)	20 (83)	
Handgrip, lbs	90.6 (42.6)	57.6 (18.8)	117.4 (37.3)	< 0.001^a^
n (%)	39 (93)	17 (100)	21 (88)	
Shoulder abduction, kg	6.2 (2.3)	4.4 (1.5)	7.4 (1.9)	< 0.001^a^
n (%)	33 (81)	13 (77)	20 (8.3)	
TST, s, median [IQR]	15.0 [12.7,17.7]	16.0 [14.8,23.5]	13.0 [12.0,15.8]	0.010^b^
n (%)	25 (61)	10 (59)	15 (63)	
**IPAQ-SF**	33 (81)	13 (77)	20 (83)	0.032^c^
Low, n (%)	7 (21)	5 (38)	2 (10)	
Moderate, n (%)	20 (61)	8 (62)	12 (60)	
High, n (%)	6 (18)	0 (0)	6 (30)	
MET, min/week, median [IQR]	1,794.0 [774.0, 2,688.0]	933.0 [469.5,1822.5]	2,393.0 [1,330.9, 3167.2]	0.001^b^
≥ 500 MET, min/week, n (%)	29 (71)	10 (59)	19 (79)	

The data are presented as the mean (SD) unless otherwise stated. R = right; L = left; n = number of; SD = standard deviation; IQR = interquartile range; TST = timed-stands test; MET = metabolic equivalent of task

^a^Student’s t-test, ^b^Mann–Whitney U-test, ^c^Chi-square test

Men with MFS demonstrated a significantly higher level of physical activity (MET min/week) than women with MFS (*p* = 0.001) (Table 2). A total of 29 patients (71%) reached the current recommendation of 500–1,000 MET min/week, of which 19 were men (Table 2).

### Results of patients with MFS compared with reference values from healthy individuals

Women and men with MFS demonstrated significant impaired maximal exercise capacity compared with reference values from healthy individuals (female sex: 112.4 [34.1] vs. 167 [12.7] W, *p* < 0.001; male sex: 165.7 [38.7] vs. 259.5 [21.6] W, *p* < 0.001) (Table 3).

**Table 3:** Tests of physical fitness in patients with Marfan syndrome compared with reference values by age

	All patients			18–29 years			30–49 years		50–69 years			
	MFS	REF	*p*	MFS	REF	*p*	MFS	REF	*p*	MFS	REF	*p*
**WOMEN**												
**Cycle test**	*n* = 13			*n* = 6			*n* = 6			*n* = 1		
Maximum, W	112.4 (34.1)	167.0	< 0.00	121.6 (38.2)	175.3 (0.5)	0.019^a^	112.7 (23.7)	164.6 (6.6)	0.002^a^	54.9 (NA⁑)	131.5 (NA⁑)	NA⁑
% of ref. value	66.7 (18.0)	(12.7)	1^a^	69.4 (21.9)			68.3 (12.9)			41.7 (NA⁑)		
**Muscle function**												
Heel lift R	*n* = 14			*n* = 6			*n* = 7			*n* = 1		
Repetitions	12.7 (5.5)	28.0 (2.8)	< 0.00	12.3 (5.4)	30.7 (0.0)	< 0.00	13.3 (6.4)	26.4 (1.4)	0.002^a^	11.0 (NA⁑)	22.6 (NA#)	NA⁑
% of ref. value	45.9 (20.1)		1^a^	40.2 (17.6)		1^a^	50.5 (24.9)			48.7 (NA⁑)		
Heel lift L	*n* = 14			*n* = 6			*n* = 7		0.005	*n* = 1	23.1	NA⁑
Repetitions	13.6 (5.9)	27.0 (2.7)	< 0.00	13.3 (6.8)	29.6 (0.0)	0.002^a^	14.0 (6.0)	25.2 (1.5)	^a^	13.0 (NA⁑)	(NA#)	
% of ref. value	51.5 (23.3)		1^a^	45.1 (22.9)			56.3 (25.8)			56.3 (NA⁑)		
Handgrip	*n* = 17			*n* = 7			*n* = 7			*n* = 3		
lbs	57.6 (18.8)	69.0 (7.9)	0.021^a^	60.3 (22.1)	72.2 (2.2)	0.239^a^	62.3 (16.2)	71.6 (7.4)	0.211^a^	40.2 (4.7)	55.8 (1.3)	0.016^a^
% of ref. value	83.5 (25.9)			84.3 (32.7)			87.8 (24.9)			71.9 (6.7)		
Shoulder abduction	*n* = 13			*n* = 6			*n* = 6			*n* = 1		NA⁑
kg	4.4 (1.5)	4.7 (NA#)	0.431^a^	4.2 (1.6)	4.7 (NA#)	0.452^a^	4.8 (1.4)	4.7 (NA#)	0.881^a^	3.0 (NA⁑)	4.7 (NA#)	
% of ref. value	92.9 (31.1)			88.9 (33.5)			101.9 (30.2)			63.8 (NA⁑)		
TST*	*n* = 10			*n* = 4			*n* = 5			*n* = 1		
s, median [IQR]	16 [14.8, 23.5]		0.007^b^	15.5 [13.5, 29.5]		0.068^b^	16 [16.9, 19.7]		0.080^b^	60.0 [NA⁑]		0.009^b^
s	22.3 (14.5)	13.3 (2.3)		19.5 (9.7)	11.3(5.2)		17.0 (2.5)	13.8 (1.1)		60.0 (NA⁑)	18.4 (NA#)	
% of ref. value	70.6 (22.3)			66.2 (21.6)			82.2 (13.7)			30.7 (NA⁑)		
**IPAQ-SF**	*n* = 13			*n* = 5			*n* = 7			*n* = 1		
Low, (%)	38	22.2		40	20		29	25		100	25	
									(NA⁑	0	46	NA⁑
Moderate, (%)	62	42.7	(NA⁑)	60	40	(NA⁑)	71	46	)	0	29	
High, (%)	0	34.9		0	40		9	29				
**MEN**												
**Cycle test**	*n* = 22			*n* = 10			*n* = 9		< 0.0	*n* = 3	212.3	0.107^a^
Maximum, W	165.7 (38.7)	259.5	< 0.00	166.4 (36.9)	266.1 (9.3)	< 0.00	172.5 (39.4)	267.8	01^a^	142.6 (48.8)	(10.6)	
% of ref. value	63.8 (14.1)	(21.6)	1^a^	62.3 (12.8)		1^a^	64.4 (14.5)	(11.4)		66.8 (21.4)		
**Muscle function**												
Heel lift R	*n* = 20			*n* = 11			*n* = 7			*n* = 2		
repetitions	21.2 (9.9)	34.4 (4.2)	< 0.00	20.6 (10.6)	37.5 (0.0)	< 0.00	18.7 (7.6)	32.3 (1.7)	0.003^a^	14.5 (7.8)	19.5 (0.0)	0.530^a^
% of ref. value	61.8 (26.9)		1^a^	55.0 (28.3)		1^a^	57.9 (22.8)			74.4 (39.9)		
Heel lift L	*n* = 20			*n* = 11			*n* = 7			*n* = 2		
repetitions	19.3 (9.2)	34.3 (4.2)	< 0.00	23.0 (11.6)	37.4 (NA#)	< 0.00	19.7 (7.8)	32.0 (NA#)	0.003^a^	16.5 (4.9)	18.8 (NA#)	0.530^a^
% of ref. value	56.6 (25.4)		1^a^	61.5 (31.1)		1^a^	61.9 (24.9)			87.8 (26.3)		
Handgrip	*n* = 21			*n* = 10			*n* = 8			*n* = 3		
lbs	117.4 (37.3)	116.6 (9.6)	0.930^a^	104.7 (23.0)	121.0 (8.4)	0.052^a^	130.1 (36.4)	118.3 (5.3)	0.423^a^	126.0 (73.0)	97.7 (13.8)	0.588^a^
% of ref. value	102.2 (40.1)			86.5 (19.0)			110.1 (35.4)			131.7 (85.3)		
Shoulder abd.	*n* = 20			*n* = 9			*n* = 7		0.132	*n* = 4	9.2	0.091^a^
kg	7.4 (1.9)	9.2 (NA#)	< 0.00	7.2 (2.0)	9.2 (NA#)	0.018^a^	7.8 (2.1)	9.2 (NA#)	^a^	6.8 (1.9)	(NA#)	
% of ref. value	80.0 (21.2)		1^a^	78.8 (21.5)			84.9 (22.8)			74 (21.1)		
TST*	*n* = 15			*n* = 8			*n* = 5			*n* = 2		
s, median [IQR]	13.0 [12.0, 15.8]		0.009^b^	13.0 [12.1, 15.7]		0.012^b^	13.0 [11.3, 14.4]	10.9 (NA#)	0.080^b^	15.5 [NA⁑]	15.7 (1.3)	0.655^b^
s	13.9 (3.2)	10.6 (2.3)		14.2 (2.9)	9.0 (0.5)		12.9 (1.9)			15.5 (7.8)		
% of ref. value	79.5 (27.8)			65.5 (10.4)			86.7 (12.3)			117.9 (68.0)		
**IPAQ-SF**	*n* = 20			*n* = 8			*n* = 8			*n* = 4		
Low, (%)	10	25.6		12.5	23		0	29		25	25	
Moderate, (%)	60	31.8	0.022^c^	75	30	0.020^c^	62.5	34	NA⁑	25	46	0.609^c^
High, (%)	30	42.5		12.5	47		37.5	37		50	29	

The data are presented as the mean (SD) unless otherwise stated. SD = standard deviation; Shoulder flex = shoulder flexion; Shoulder abd. = shoulder abduction; TST = timed-stands test; NA = not available

⁑Insufficient data for analysis

#SD not available

*Non-normally distributed reference values are presented as the mean (SD), % of reference values are based on the mean

^a^One-sample t-test, ^b^One-sample Wilcoxon signed rank test, ^c^Chi-square test

**Fig. 2:**
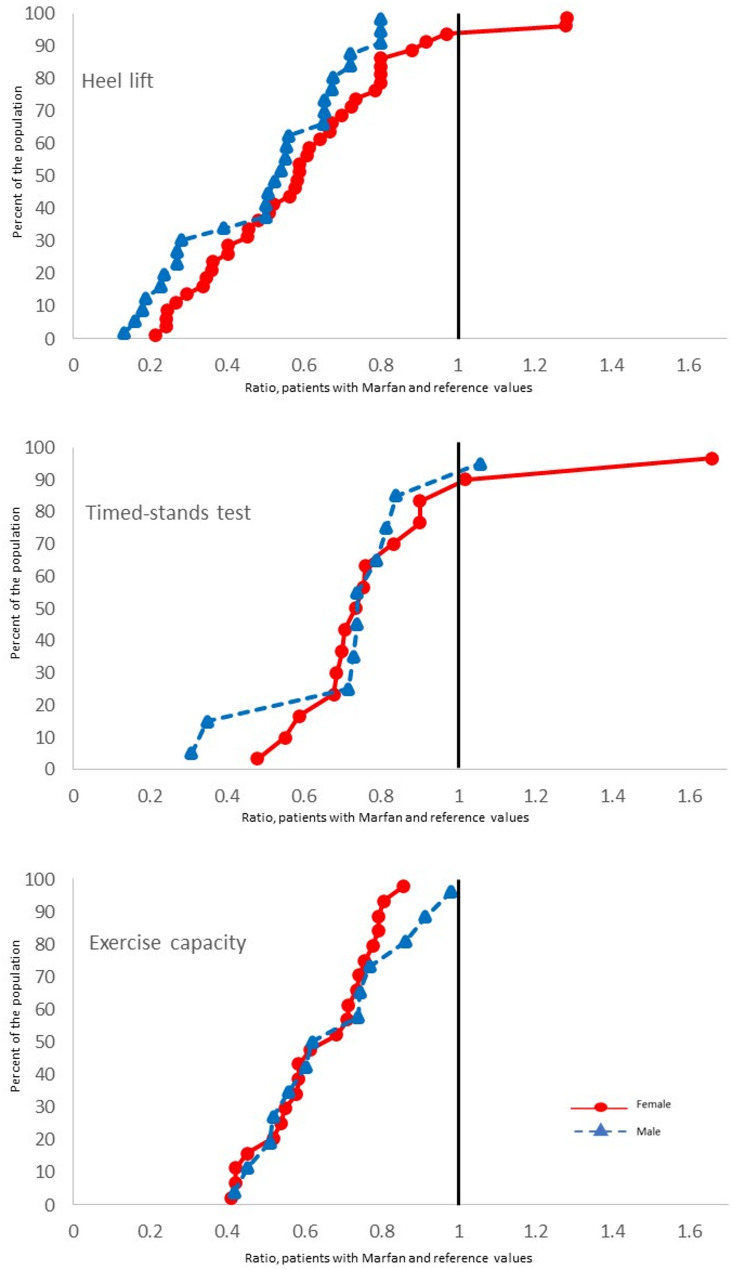
Cumulative distribution (1.0 = reference value) in patients with MFS in relation to reference values. MFS = Marfan syndrome

**Fig. 3:**
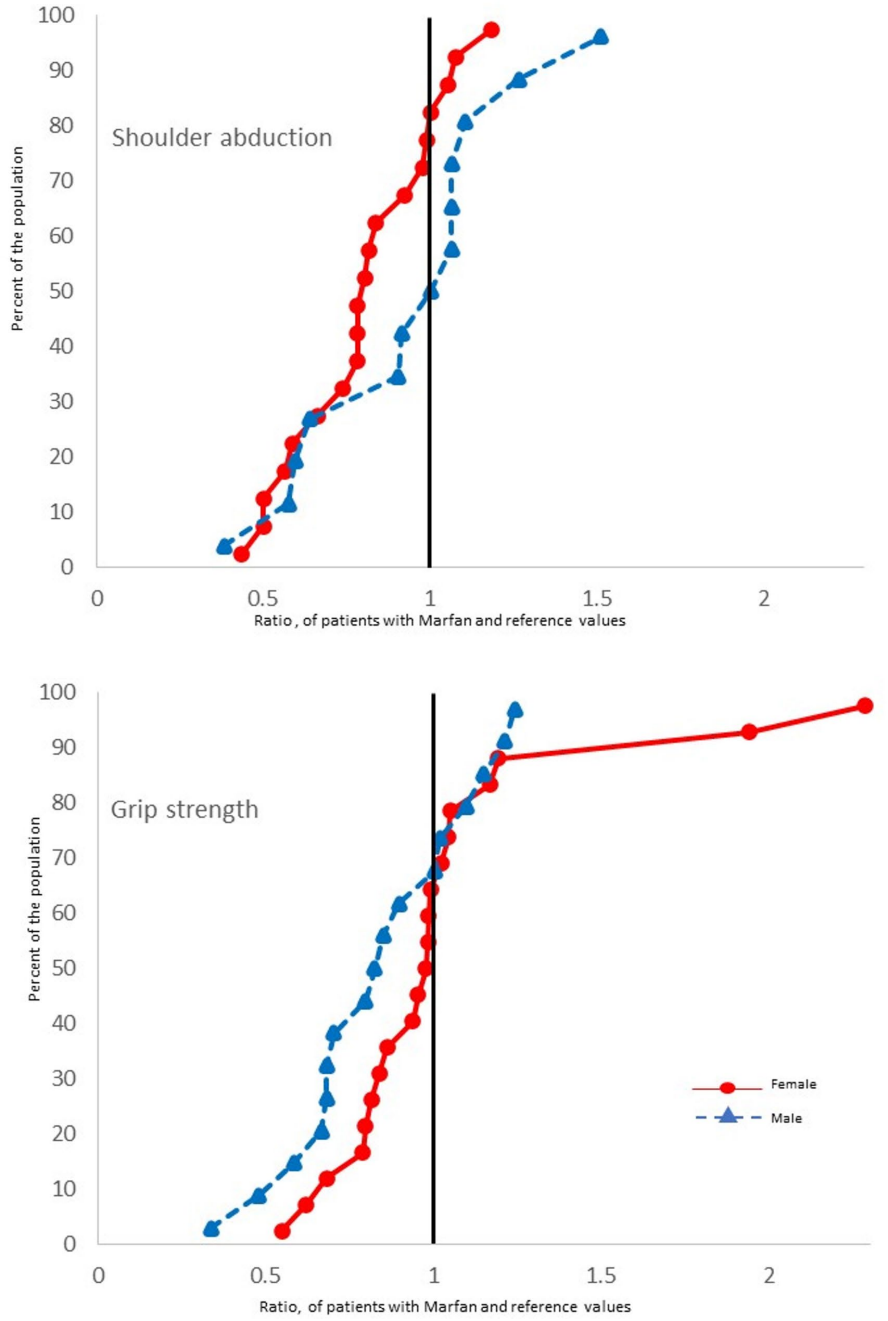
Cumulative distribution (1.0 = reference value) of isometric tests in patients with MFS in relation to reference values. MFS = Marfan syndrome

Both women and men with MFS demonstrated significant impairment in several muscle function tests compared with reference values from healthy individuals (Table 3). Women with MFS demonstrated significant impairments in right heel lift (MFS: 12.7 [5.5] vs. reference: 28.0 [2.8] repetitions; *p* < 0.001), left heel lift (MFS: 13.6 [5.9] vs. reference: 27.0 [2.7] repetitions; *p* < 0.001), handgrip strength (MFS: 57.6 [18.8] vs. reference: 69.0 [7.9] lbs; *p* = 0.021), and TST (*p* = 0.007). Men with MFS demonstrated significant impairment in right heel lift (MFS: 21.2 [9.9] vs. reference: 34.4 [4.2] repetitions; *p* < 0.001), left heel lift (MFS: 19.3 [9.2] vs. reference: 34.3 [4.2] repetitions; *p* < 0.001), shoulder abduction (MFS: 7.4 [1.9] vs. reference: 9.2 (SD not available) kg; *p* < 0.001), and TST (*p* = 0.009) (Table 3). The cumulative distribution regarding exercise capacity and tests of isoinertial muscle function in men and women with MFS in relation to healthy reference values are displayed in Fig. 2, and isometric tests displayed in Fig. 3.

No women with MFS reached the high physical activity level, while 34.9% of healthy individuals did. Among men with MFS aged 18–29 years, 12.5% demonstrated a high level of physical activity compared with 47% of healthy individuals. Among men with MFS aged 50–69 years, 50% demonstrated high physical activity compared with 29% of healthy individuals (Table 3).

## Discussion

In the present study, women and men with MFS had a significantly lower exercise capacity than healthy individuals according to reference values [16]. The results are in line with the results of Giske et al. who found a reduced peak oxygen uptake by 20**–**30% [11]. The reason for this is likely multifactorial, including a reduced heart rate reserve caused by beta-blocker use [30], which was present in 54% of patients in the present study; a lifelong fear of exercise, which limits physical activity [8]; and inexperience with exercise and reduced confidence when performing exercise [31]. Height is the most prominent factor for high exercise capacity on an ergometer cycle [16]. The mean height of women in Sweden is 166 cm, and the mean height of men is 180 cm [32]. However, patients with MFS in the present study were taller (women: 177 cm; men: 195 cm) than the average Swedish population. This may explain why the exercise capacity of the patients in the present study was higher. A Norwegian study from 2003 which assessed knee flexion and extension in young adults (*n* = 17) reported that isokinetic peak torque was reduced only at the highest velocity [11]. Furthermore, a previous study from 2007 included women (*n* = 21) with no previous aortic surgery and reported a decreased isometric and isokinetic muscle strength in both the quadriceps and hamstrings muscle [12]. The results of the present study showed that both men and women with MFS had decreased muscle function regarding left and right heel lifts, and timed-stands test, as well as reduced handgrip strength for women and shoulder abduction for men. The present study could thereby present results of muscle function for the upper extremity in patients with MFS which has not previously been reported.

Both exercise capacity and skeletal muscle mass decline with age [33, 34]; therefore, a physiological approach was taken when dividing patients with MFS into different age groups. In the healthy population, individuals aged 18–29 years consistently had higher values for exercise capacity [16] and muscle function [23, 25, 35] than the other age groups. This does not conform directly with the test results of patients with MFS, which showed that in many cases, the 30–49-year age group demonstrated the best test results. The results of this study were also not in line with the negative correlation between aging and maximal oxygen consumption (VO_2max)_, and between aging and muscle mass, described previously [33, 34]. In the healthy population, VO_2max_ starts to decline at around 30 years of age in both sexes and deteriorates more rapidly thereafter. The difference between women and men in VO_2max_ is greater at a younger age, and the difference decreases among older age groups, which can be explained by men losing more fat-free mass than women with age [33]. Total muscle mass starts to decline around 25–30 years of age in both sexes, with a more rapid decline from the age of 50 years. On average, 40% of total muscle mass has been lost by 80 years of age [34]. It is therefore somewhat surprising that 30–49-year-olds with MFS in the present study demonstrated better exercise capacity and muscle function than the 18–29-year age group. The reason behind this is unknown, but it could be due to the small sample size of patients with MFS.

Fatigue and pain are two factors that could have negatively affected the test results, both of which are common in patients with MFS [5, 6]. According to Bathen et al. [5], fatigue affects the daily lives of patients with MFS, as well as their motivation to perform tasks. A positive outcome of that study was that fewer patients with MFS reported exercise-induced fatigue. Speed et al. [6] described that chronic pain is common in patients with MFS. Pain can occur in multiple locations of the body, frequently involving the back. The same study also showed that the experience of pain leads to symptoms such as stiffness, difficulty walking, muscle spasm, and muscle weakness, which could have affected testing in that study. Speed et al. [6] also concluded that even though pain is common in patients with MFS, few patients are offered help with pain management.

In the present study, men with MFS demonstrated a higher level of physical activity (MET min/week) than women with MFS, with 30% of men demonstrating a high level of physical activity compared with none of the women. In the healthy population, men also reported a somewhat higher rate of high physical activity than women (42.5% vs. 39.4%). However, activity patterns in women with MFS showed that none achieved a high level of activity, while 40% of healthy women did. This may imply an altered activity pattern in women with MFS. The results are in line with a previous study reporting that women with MFS were significantly less active at the vigorous activity level than controls [12]. The majority of patients of both sexes were within the limits of the current recommendation, with 10 women (59%) and 19 men (79%) reaching ≥ 500 MET min/week [29]. A systematic review from 2011, which assessed the validity of the IPAQ-SF, found a low median correlation of 0.29 when comparing objectively with subjectively measured physical activity. Furthermore, it is common to overestimate when self-reporting the level of physical activity [33]. The self-reported level of physical activity in the present study may therefore be somewhat overestimated [36], especially for patients with a significantly reduced physical capacity compared with reference values from the healthy population.

A systematic review from year 2022 which assessed physical activity as a future therapy for patients with MFS reported on the positive impact of exercise on subjects with MFS observed in animal studies [7]. Two separate controlled trials on mice with MFS noted a decrease in aortic diameter growth rate after 5 months of moderate-intensity isoinertial exercise [37, 38]. These results are highly interesting if they are implementable to patients with MFS, for whom exercise is often restricted [7] and aortic root pathology is the leading cause of death [4]. During isoinertial exercise, peripheral blood pressure increases, while central blood pressure remains steady [39]. During isometric exercise, however, peripheral systolic blood pressure increases slightly, while central blood pressure increases significantly [39]. This physiological response creates mechanical stress on the aortic wall [40]. According to a review from 2020, both isometric and isoinertial resistance exercises involving small muscle groups result in similar elevations in systolic and diastolic blood pressure [41]. However, the blood pressure response to isometric and isoinertial resistance exercise with large muscle groups is undetermined and requires further research [41]. There is little to no evidence suggesting either benefits or disadvantages of exercising at low, moderate, or high intensity with MFS [7, 42]. Further research evaluating physical capacity and level of physical activity in adults with MFS, with a larger study population, as well as research evaluating if the negative correlation between aging, VO_2max_, and muscle mass is implementable to patients with MFS is desirable.

### Study strengths & limitations

This study has some particular strengths. First, MFS is a rare disorder, and in the present study, 41 patients with MFS were included. Few studies involve patients with MFS; therefore, the evaluation of physical fitness and level of activity in this study of patients with MFS is a strength. Second, a standardized protocol was used for testing. The test order was predetermined, and standardized instructions were used to test the starting positions and execution. Furthermore, the equipment used to measure exercise capacity was routinely calibrated every 6 months.

The present study also had the following limitations. First, the reference values used for the muscle function tests were based on the Swedish population and correspond well to the determined age groups; however, there are exceptions. For unilateral isometric shoulder abduction, reference values were missing for each age group; thus only sex comparisons could be made. Moreover, reference values for unilateral isoinertial shoulder flexion were not available. Second, the sample size of the present study is comparable to those of other studies in patients with MFS regarding physical fitness [7, 8]. However, stratifying the patients by age meant that the sample size in each respective age group was small (the 50–69-year age group for both female and male patients included only three women and four men, respectively), impacting the ability to detect statistical differences. No power or sample size calculations of the population were performed as no previous studies were found which evaluated effect size of the included tests of physical fitness in patients with MFS. Third, the reasons why some patients did not perform tests of physical fitness (*n* = 6) are unknown. It is possible that because the test battery used in the clinic was extensive, other tests may have been prioritized, or the patient or the assessor may have lacked time. Nevertheless, a drop-out analysis of these patients showed no significant differences in age, sex, or NYHA functional classification compared with the included patients. Fourth, the inclusion criteria required only one test to be completed for the patient to be included, which resulted in missing values for the tests that was not performed; other patients performed the entire test battery. The TST was not included in the test battery until 2013; therefore, fewer patients may have performed this test. Finally, we could potentially have missed patients who were not diagnosed with MFS according to the specific ICD 10 Q874 diagnosis and who were only diagnosed with a specific aortopathy.

## Conclusions

In this study, patients with MFS demonstrated significant impairment in exercise capacity and muscle function compared with healthy individuals. Women with MFS demonstrated greater impairment in both exercise capacity and muscle function than men with MFS, which is in line with sex differences in the reference values of healthy individuals. Furthermore, 71% of patients with MFS reached the general recommendation for physical activity. In the clinical setting, tests of physical fitness may be of importance in patients with MFS to be able to prescribe individualized exercise. Additional research is needed to assess whether individually prescribed exercise regimes can improve physical fitness in adults with MFS.

## Electronic supplementary material

Below is the link to the electronic supplementary material.

Supplementary Material 1

## Data Availability

Research data is confidential due to ethical and legal reasons. Permission to use data is only that which has been given ethical approval by the Swedish Ethical Review Authority. Data are therefore only available from the corresponding author on reasonable request and with a valid ethical approval.
