# Supplementary material for: Physical fitness and level of physical activity in adult patients with Marfan syndrome
Source: Orphanet J Rare Dis. 2025 Jul 7;20:346. doi: 10.1186/s13023-025-03869-z (PMC12235886; doi:10.1186/s13023-025-03869-z)
Supplement: Supplementary file 1 — Supplementary Material 1 [file 13023_2025_3869_MOESM1_ESM.docx]

**Additional file 1**

1. **Results**

Supplementary Figure 1 depicts participation in tests of physical fitness. There was a significant difference in shoulder flexion in women with MFS between the age groups (18–29 years: 22 [9.7] kg, 30–49 years: 30 [9.2] kg, 50–69 years: 10 [3.1] kg; p = 0.016) (Supplementary Table 1).

**
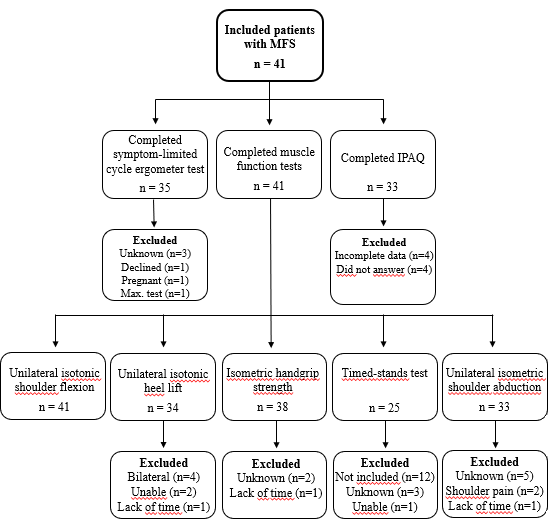
**

**Supplementary Figure 1. Flow diagram of participation in tests of physical fitness**

MFS = Marfan syndrome; n = number of; IPAQ = International Physical Activity Questionnaire

**Supplementary Table 1.** Tests of physical fitness and level of physical activity in patients with MFS by sex and age group

|  | **Female 18–29 years**  **n = 7** | **Female 30–49 years**  **n = 7** | **Female 50–69 years**  **n = 3** | **p** | **Male 18–29 years**  **n = 11** | **Male 30–49 years**  **n = 9** | **Male 50–69 years**  **n = 4** | **p** |
| --- | --- | --- | --- | --- | --- | --- | --- | --- |
| **Cycle test**  Submaximum, W  n (%) | 92 (35.6)  6 (86) | 88 (25.1)  6 (86) | 33 (NA⁑)  1 (33) | 0.247^a^ | 122 (30.8)  10 (91) | 133 (34.7)  9 (100) | 97 (34.6)  3 (75) | 0.282^a^ |
| **Muscle function** |  |  |  |  |  |  |  |  |
| Shoulder flexion, repetitions  n (%) | 22 (9.7)  7 (100) | 30 (9.2)  7 (100) | 10 (3.1)  3 (100) | 0.016^a^ | 33 (15.5)  11 (100) | 45 (21.4)  9 (100) | 39 (28.0)  4 (100) | 0.386^a^ |
| Heel lift R, repetitions  n (%) | 12 (5.4)  6 (86) | 13 (6.4)  7 (100) | 11 (NA⁑)  1 (33) | 0.918^a^ | 21 (10.6)  11 (100) | 19 (7.6)  7 (78) | 15 (7.8)  2 (50) | 0.691^a^ |
| Heel lift L, repetitions  n (%) | 13 (6.8)  6 (86) | 14 (6.0)  7 (100) | 13 (NA⁑)  1 (33) | 0.977^a^ | 23 (11.6)  11 (100) | 20 (7.8)  7 (78) | 17 (5.0)  2 (50) | 0.637^a^ |
| Handgrip, Ibs  n (%) | 60.3 (22.1)  7 (100) | 62 (16.2)  7 (100) | 40.2 (4.7)  3 (100) | 0.217^a^ | 104.7 (23.0)  10 (91) | 130.1 (36.4)  8 (89) | 125.9 (73.0)  3 (75) | 0.342^a^ |
| Shoulder abduction, kg  n (%) | 4 (1.6)  6 (86) | 5 (1.4)  6 (86) | 3 (NA⁑)  1 (33) | 0.517^a^ | 7 (2.0)  9 (82) | 8 (2.1)  7 (78) | 7 (2.0)  4 (100) | 0.714^a^ |
| TST, s, median [IQR]  n (%) | 16 [13.5, 29.5]  4 (57) | 16 [14.9, 19.7]  5 (71) | 60 [NA⁑]  1 (33) | 0.275^b^ | 13 [12.1, 15.7]  8 (73) | 13 [11.3, 14.4]  5 (56) | 16 [NA⁑]  2 (50) | 0.845^b^ |
| **IPAQ-SF**  Low, n (%)  Moderate, n (%)  High, n (%) | 5 (71)  2 (40)  3 (60)  0 (0) | 7 (100)  2 (29)  5 (71)  0 (0) | 1 (33)  1 (100)  0 (0)  0 (0) | 0.388^b^ | 8 (73)  1 (12.5)  6 (75)  1 (12.5) | 8 (89)  0 (0)  5 (62.5)  3 (37.5) | 4 (100)  1 (25)  1 (25)  2 (50) | 0.358^b^ |
| MET min/week, median [IQR] | 1506 [488, 2080] | 933 [542, 1737] | 132 [NA⁑] | 0.220^b^ | 1593 [825, 2770] | 2586 [1835, 3951] | 2713 [1116, 6079] | 0.379^b^ |

The data are presented as the mean (SD) unless otherwise stated. L = left; R = right; n = number of; SD = standard deviation; IQR = interquartile range; Handgrip = Isometric handgrip strength; TST = timed-stands test; CI = confidence interval; NA = not available; MET = metabolic equivalent of task

⁑ Insufficient data for adequate analysis.

^a^One-way analysis of variance, ^b^Kruskal–Wallis test
